# Innovations en vaccinologie: enjeux et perspectives pour l’Afrique

**DOI:** 10.11604/pamj.2017.26.235.11527

**Published:** 2017-04-25

**Authors:** Doudou Diop, Melvin Sanicas

**Affiliations:** 1 Centre de Recherche Biomédicale Espoir Pour La Santé (CRB-EPLS), Saint-Louis, Sénégal; 2 Sanofi Pasteur, Asie, Japon et région du Pacifique, Singapour

**Keywords:** Innovations, vaccins, santé publique, enjeux, perspectives, Afrique, Innovations, vaccines, public health, challenges, perspectives, Africa

## Abstract

La vaccination est incontestablement l’une des interventions de santé publique les plus efficaces et les plus rentables qui soient. Les vaccins continuent de révolutionner notre capacité à prévenir les maladies et à améliorer la santé. Avec toutes les avancées technologiques, nous sommes en mesure d’étendre les avantages des vaccins à plus de gens et de fournir une meilleure protection contre les maladies infectieuses mortelles. Toutefois, avec le développement incessant de nouvelles souches microbiennes à travers le monde, la recherche en vaccinologie se doit d’innover continuellement. D’énormes progrès ont été réalisés pour améliorer la couverture vaccinale et introduire de nouveaux vaccins en Afrique. De nouveaux types de vaccins associés à des outils de vectorisation, d’administration et de délivrance spécifiques mais aussi des adjuvants susceptibles de moduler finement la réponse immunitaire sont attendus dans le futur. En Afrique, il est nécessaire de développer une approche régionale afin de répondre efficacement aux nombreux défis. Une meilleure information, la formation des personnels de santé en vaccinologie et des recherches bien ciblées sont les clés des futurs accomplissements dans le domaine.

## Abstract (en)

Immunization is undoubtedly one of the most effective and most cost-effective health interventions. Vaccines continue to revolutionize our ability to prevent diseases and improve health. With all the technological advances, we are able to extend the benefits of vaccines to more people and to provide better protection from life-threatening infectious diseases. Nevertheless, thanks to the unceasing implementation of novel microbial strains on a worldwide basis, research in vaccinology must innovate continuously. African countries have made great progress in increasing routine immunization coverage rates and in introducing newly developed vaccines. New types of vaccines associated with vectorization, administration and specific licensing tools as well as with adjuvants designed to finely modulate immune responses are expected to be achieved in the near future. African countries need to work to develop a regional approach to respond effectively to the many challenges. Providing better information, implementing healthcare personnel vaccinology training programs and well targeted research projects are the keys to future achievements in the field.

## Introduction

La vaccination reste le moyen le plus efficace et le moins coûteux de se protéger contre les maladies infectieuses et certains types de cancers. Elle permet de sauver 2 à 3 millions de vies chaque année dans le monde [1]. Elle poursuit ainsi deux objectifs complémentaires : procurer un bénéfice individuel, en s’opposant aux effets pathogènes des agents infectieux et assurer un bénéfice collectif de santé publique, en limitant la circulation et la transmission de ces agents. Outre les résultats de santé, les vaccins offrent un certain nombre d’avantages, notamment les frais médicaux évités et la baisse de l’absentéisme dans les lieux de travail causé par des maladies évitables par la vaccination. Ces économies profitent aux familles, aux communautés et aux nations en ce qu’elles permettent d’améliorer l’éducation, de renforcer la croissance économique et de réduire la pauvreté [2]. Une étude a indiqué qu’une augmentation de la couverture vaccinale dans les pays éligibles au soutien de GAVI pourrait assurer un retour sur investissement de 18 % d’ici à 2020 [3, 4]. La vaccination touche donc de multiples dimensions sociétales : scientifiques, médicales, sociales, économiques et politiques. La science qui englobe tous ces aspects liés à la vaccination est appelée la vaccinologie.

Le terme innovation apparut avec la révolution industrielle [5]. Dans le secteur médical, l’action d’innover correspond à l’introduction et la diffusion de produits, de techniques et de nouvelles méthodes d’organisation permettant d’améliorer la santé et lutter contre la maladie [5]. Depuis leur invention, les vaccins sont un des moteurs de l’innovation, tant au niveau de la recherche et développement (R&D), des politiques sanitaires, que de la production industrielle. De nombreux vaccins ou conjugaisons de vaccins sont aujourd’hui à la disposition de la population. En Afrique, d’importants progrès ont été notés cette dernière décennie dans le domaine de la vaccinologie [6]. Toutefois, avec le développement incessant de nouvelles souches microbiennes à travers le monde, la recherche en vaccinologie se doit d’innover continuellement. En Afrique en particulier, le renforcement des capacités intrinsèques et l’adoption d’une approche systémique du développement des technologies de la santé représentent un enjeu de toute première importance pour la santé publique et son économie.

### Grandes réalisations au cours de la dernière décennie

D’importantes réalisations, allant de l’introduction de nouveaux vaccins à l’usage de nouvelles techniques de contrôle de la chaine de froid, en passant par l’amélioration des schémas de vaccination pour certains vaccins déjà disponibles dans les programmes d’immunisation, ont été enregistrées en Afrique au cours de la dernière décennie.

### Introduction du vaccin contre le méningocoque du sérogroupe A

Le méningocoque du sérogroupe A a été l’une des causes les plus courantes de méningite en Afrique subsaharienne, causant de graves épidémies saisonnières qui ont décimé des milliers de personnes et handicapé beaucoup d’autres. Grâce à un partenariat mondial unique, un vaccin conjugué contre le méningocoque du sérogroupe A abordable (moins de 0,50$ US par dose) et efficace a été développé spécifiquement pour l’Afrique [7]. Le vaccin MenAfriVac^®^ a été introduit à travers des campagnes de vaccination de masse avec le soutien de GAVI, et a été administré à plus de 220 millions d’enfants et d’adultes dans 16 pays de la « ceinture africaine de la méningite » entre 2010 et 2015 [8]. Le méningocoque du sérogroupe A a pratiquement été éliminé de la région, ce qui constitue un énorme succès en matière de santé publique. En 2013, seulement quatre cas de méningite A confirmés en laboratoire ont été recensés dans les 26 pays de la « ceinture africaine de la méningite » [9]. Toutefois, pour éviter la résurgence de la maladie les programmes de vaccination systématique doivent maintenir une couverture élevée [10]. Le projet vaccins méningite (MVP) reste un modèle intéressant par lequel un vaccin est développé pour répondre aux besoins spécifiques d’une population avec la participation active des scientifiques de ces pays.

### Introduction du vaccin contre le papillomavirus humain

Le cancer du col de l’utérus est la principale cause de décès des suites de cancer chez les femmes dans les pays en développement du fait du manque d’accès aux services de dépistage et de traitement. Deux vaccins sont maintenant disponibles dans la plupart des pays développés et offrent une protection contre les souches de papillomavirus humain (VPH) qui causent environ 70 % des cas de cancer du col de l’utérus. Avec le soutien de GAVI, huit pays africains ont maintenant introduit les vaccins anti-VPH dans leurs programmes de vaccination systématique et 21 autres pays mènent des projets de démonstration sur les vaccins avant une utilisation généralisée [11].

### Introduction du vaccin contre le rotavirus

Le rotavirus est la principale cause de diarrhée sévère chez les enfants à travers le monde [12]. Contrairement à d’autres causes de diarrhée, le rotavirus ne peut pas être prévenu en améliorant la qualité de l’eau et en respectant simplement les règles d’hygiène, et il ne peut pas être traité avec des antibiotiques. Les vaccins contre le rotavirus sont alors considérés comme l’une des interventions les plus rentables pour prévenir les maladies diarrhéiques et les décès imputables à la diarrhée [13]. Depuis 2015, 30 pays africains, dont beaucoup enregistraient les taux de mortalité infantile les plus élevés sur le continent, ont introduit les vaccins contre le rotavirus [14].

### Introduction d’une deuxième dose de vaccin contre la rougeole (MCV2)

On estime que 48 000 cas de décès liés à la rougeole sont survenus en Afrique en 2014, ce qui représente 42 % des décès imputables à cette maladie dans le monde [15]. Le virus de la rougeole étant très contagieux, le contrôle exige une couverture de plus de 95% avec deux doses de vaccin administrées dans le cadre de la vaccination systématique ou des activités de vaccination supplémentaires dans tous les districts sanitaires [16]. A ce jour, près de la moitié des pays en Afrique ont intégré le MCV2 dans leurs programmes de vaccination systématique avec toutefois des taux de couverture qui restent encore faibles [17]. Certains pays ont même introduit simultanément le vaccin contre la rubéole. Ces efforts doivent être renforcés en vue de l’élimination de la rougeole dans la région africaine, si l’on sait que le comité régional OMS de l’Afrique a fixé l’objectif de l’élimination de la maladie d’ici à 2020.

### Vaccination contre l’hépatite B à la naissance

L’efficacité du vaccin contre l’hépatite B dans la prévention du cancer du foie viro-induit est aujourd’hui scientifiquement démontrée par de nombreuses études, notamment asiatiques [18]. Le vaccin permet de diminuer considérablement les risques de développer plus tard une lésion cancéreuse potentiellement mortelle. Récemment, plusieurs pays africains ont introduit une dose de vaccin à la naissance portant le total des doses administrées à quatre qui est le schéma vaccinal qui confère une plus grande protection contre la maladie.

### Élimination du tétanos

Même s’il est impossible d’éradiquer le tétanos (puisque les spores du tétanos sont répandues dans l’environnement), il existe des interventions et des stratégies très efficaces et essentielles pour contrôler la maladie, notamment la vaccination des mères et des techniques hygiéniques pour l’accouchement et le soin du cordon ombilical. En 2014, 77 % des enfants africains ont été protégés contre le tétanos néonatal grâce à la vaccination maternelle [19]. Cet effort doit être poursuivi dans tous les pays africains afin d’atteindre l’objectif d’élimination du tétanos maternel et néonatal (définie comme l’enregistrement de moins d’un cas pour 1000 naissances vivantes) dans chaque district.

### Elimination de la poliomyélite

Le 11 août 2015 marque la date d’une première année écoulée depuis la détection du dernier cas d’infection par un poliovirus sauvage sur l’ensemble du continent africain [20] ce qui constitue une étape importante dans la lutte contre cette maladie et reflète les contributions des dirigeants politiques, des professionnels de la santé, et des communautés à travers le continent. Malheureusement, en août 2016, le gouvernement du Nigeria a signalé que deux enfants avaient été paralysés par la poliomyélite. Cela a conduit à une nouvelle réponse d´urgence dans un pays que l´OMS avait déclaré «indemne de poliomyélite» en 2015. Pour assurer la pérennité des succès obtenus dans la lutte contre la poliomyélite en Afrique, il y’a un certain nombre de défis à relever notamment l’amélioration de la couverture du vaccin antipoliomyélitique oral (VPO), la surveillance soutenue et le maintien de la capacité de riposte face aux flambées, et l’introduction du vaccin antipoliomyélitique inactivé (IPV) dans les systèmes de vaccination systématique qui doit être poursuivie.

### Amélioration des méthodes de gestion de la température des vaccins et de la logistique

La réussite de la gestion de la chaîne du froid et de la logistique nécessite une attention à de nombreuses considérations. Récemment des avancées majeures ont été notées dans ce domaine en Afrique. Le vaccin MenAfriVac^®^ a été utilisé pour la première fois à l’extérieur de la chaîne de froid lors d´une campagne de vaccination à grande échelle dans le district de Banikoara au Bénin [21, 22]. Le vaccin est resté stable à l´extérieur de la chaîne du froid à des températures ne dépassant pas 40°C pendant au plus quatre jours. La surveillance active des événements indésirables suite à l´immunisation a indiqué qu’il n´y avait pas eu d´augmentation des manifestations adverses post-immunisation (MAPI) avec la pratique de la chaîne à température contrôlée (CTC) comparativement aux zones contrôles, et qu´aucun évènement indésirable grave n´a été signalé. Actuellement, le MenAfriVac^®^ est le seul vaccin homologué par l’OMS et étiqueté en vue d’une utilisation en CTC. Cette pratique offre plusieurs avantages dans les zones difficiles d’accès ou celles où la chaine de froid est faible. Pour garantir un transport sécurisé des nouveaux vaccins, le programme élargi de vaccination tunisien a étudié avec le projet Optimize la possibilité d´utiliser des packs PCM (à changement de phase) plutôt que des accumulateurs de froid pour le stockage à réfrigération passive [23]. Ces PCM constituent une solution idéale, car ils gèlent à une température adaptée au stockage des vaccins (5°C) et peuvent entrer en contact direct avec les vaccins thermosensibles, ce qui permet de stocker des vaccins dans toutes les zones du conteneur, en outre ils assurent un refroidissement suffisant pour une journée de travail [23]. Au Sénégal, des systèmes d’entrepôts mobiles avec plusieurs conteneurs ont été testés dans la région médicale de Saint-Louis. Les conteneurs sont refroidis à l´aide de PCM qui ont été réfrigérés à une température adaptée au stockage des vaccins pour une période donnée [23]. Ce système de réfrigération apporte une garantie de stabilité lorsque l´alimentation électrique fonctionne par intermittence. Plus spécifiquement, la technologie Sure Chill^®^, qui offre une durée de maintien en température inédite, permet d´éviter l´utilisation de générateurs d´appoint lorsque le réseau fournit de l´électricité au moins 8 heures par jour [23].

### Enjeux et perspectives

L’innovation technologique initiée pendant la première décade du XXIe siècle dans la découverte et la formulation d´antigènes ainsi que la connaissance plus profonde des réponses immunitaires humaines ouvrent la voie au développement de nouveaux vaccins et offre d’importantes promesses en santé publique pas seulement en Afrique mais à l’échelle mondiale. Parmi les approches innovantes, trois voies sont particulièrement prometteuses : la génétique inverse et la vaccinologie inverse, les vaccins recombinants vivants multivalents et les vaccins « ADN » [24 - 26]. La vaccinologie inverse est maintenant utilisée pour produire rapidement de nouvelles souches vaccinales et pour exploiter des génomes entiers dans la recherche d´antigènes [25, 26]. Les candidats vaccins recombinants vivants multivalents sont des micro-organismes rendus capables par recombinaison génétique de produire simultanément les antigènes vaccinants de plusieurs pathogènes différents [24]. Ces constructions pourraient permettre la fabrication de vaccins combinés très performants. Plus récemment, la recherche de vaccins à base de matériel génétique s’est développée. Par l’injection de fragments d’ADN appropriés il est possible d’obtenir des réponses immunes efficaces contre les microorganismes correspondants. Ces vaccins « ADN » auraient plusieurs avantages, en particulier de faciliter l’utilisation d’antigènes rares ou instables [24]. La conception rationnelle des adjuvants est devenue possible à la suite de la découverte des récepteurs qui reconnaissent les modèles microbiens et conduisent à l´activation des cellules dendritiques [25, 26]. Des progrès récents dans la connaissance des mécanismes fondamentaux de la réponse immunitaire permettent d’élargir le principe de la vaccination à différentes pathologies non infectieuses notamment les cancers. L’Afrique a un rôle fondamental à jouer dans ces processus d’innovations non pas en termes d’investissements financiers mais plutôt par une contribution scientifique, technique et administrative. Dans le domaine de la R&D et du développement industriel, il faudrait promouvoir sur le continent une démarche partenariale public-privé, créer des pôles de compétitivité, et proposer des plates-formes nationales et internationales de développement et de suivi (comportant en particulier des réseaux de surveillance et de larges bases de données médico-administratives) permettant une évaluation clinique pré et post-enregistrement à large échelle des nouveaux vaccins ainsi que leur impact en termes de morbidité, de recours aux soins, mais aussi économique. Sur le plan académique, il est nécessaire de créer un réseau international d’experts en vaccinologie, incluant sciences fondamentales, sciences économiques, sociales et politiques, impliqués tant dans les aspects fondamentaux, que dans la formation et l’évaluation des politiques de vaccination. La formation en vaccinologie des médecins, des infirmiers et autres professionnels de santé est tout aussi nécessaire. Même si les maladies infectieuses, l´immunologie, la microbiologie et la santé publique sont couvertes par les programmes de formation médicale, les regrouper sous la rubrique vaccinologie aiderait à concentrer plus l´attention sur l´importance de la vaccination dans la pratique médicale. Les initiatives déjà prises dans ce sens telles que le cours annuel africain de vaccinologie (AAVC) de l’Université du Cap en Afrique du Sud, la sélection des médecins et scientifiques africains aux cours avancés de vaccinologie (ADVAC) de la Fondation Mérieux en partenariat avec l’Université de Genève, et le Master de vaccinologie de l’Université de Sienne, Italie en partenariat avec Novartis Vaccines (actuellement GSK), doivent être encouragées et soutenues. D’autre part, il est nécessaire d’adopter une approche régionale et globale impliquant tous les partenaires, décideurs, professionnels de santé, industriels, et le public lui-même afin d’améliorer la perception de la valeur des vaccins et l’adoption plus aisée des politiques vaccinales par les populations.

La vaccination est une intervention de santé simple avec des impacts économiques importants. Récemment, une étude publiée dans le bulletin de l´OMS a montré que l´utilisation d´une approche CTC peut réduire les coûts liés à la chaîne du froid de 50% lors des campagnes. Pour les campagnes MenAfriVac^®^ entre 2014 et 2016, les économies se traduiraient à plus de 12 millions de dollars [21]. Pour faire faire plus d’économies aux pays africains, une autre solution possible est la production de vaccins pré-qualifiés par l´OMS en Afrique. Mais pour atteindre cet objectif, il faudrait améliorer l’environnement réglementaire en passant par une harmonisation des procédures d’autorisation d’essais cliniques et de mise sur le marché des vaccins. D´après le Council on Health Research for Development (COHRED), presque tous les vaccins homologués par la GAVI sont produits en Europe, en Amérique du Nord, en Amérique latine et en Asie [27]. Dans une lettre ouverte, le COHRED déclarera, que “le développement durable pourrait être largement amélioré en Afrique si la production et la réglementation locales des vaccins étaient incluses dans les objectifs mondiaux”. Il existe sur le continent un seul producteur de vaccins ayant été approuvé (´pré-qualifié´) par l´OMS, à savoir l´Institut Pasteur de Dakar, au Sénégal, qui fabrique des vaccins contre la fièvre jaune. L´Afrique du Sud est toutefois en train de créer l´Institut Biovac et la Tunisie accroît sa capacité à produire des vaccins. D´après le COHRED, d´autres pays africains, tels que le Kenya et l´Ouganda, disposent également d´établissements qui pourraient, avec un peu d´aide, devenir des producteurs de qualité [27].

Pour une bonne prise en charge des systèmes d’approvisionnement, les dirigeants africains sont invités à investir dans les chaînes d’approvisionnement de nouvelle génération qui changent fondamentalement la façon dont les vaccins sont gérés et sont fournis aux communautés. Le système CTC permet en outre d’accroître la couverture vaccinale dans les secteurs où l´accès est un problème et l´électricité n´est pas fiable. La couverture impressionnante obtenue au Bénin lorsque MenAfriVac^®^ a été utilisé en dehors de la chaîne du froid ouvre la voie à de futures campagnes dans d´autres régions à géographie difficile, y compris les pays où le vaccin sera administré plus tard. Trouver des solutions pour réduire les coûts et les difficultés logistiques d´atteindre les personnes vivant dans des régions éloignées supprime une contrainte majeure à la couverture universelle des vaccins au-delà de MenAfriVac^®^. En effet, une approche similaire est explorée avec les fabricants d´autres vaccins, tels que la vaccination contre la fièvre jaune ou le choléra oral [28]. Si des progrès importants ont été faits dans la lutte contre le cancer du foie à travers la vaccination contre le virus de l’hépatite B, il reste en cours du chemin à parcourir dans la lutte contre les cancers du col de l’utérus, la plupart des pays africains ayant introduit le vaccin anti-VPH que tout récemment et à des échelles sous-nationales le plus souvent. Sur ce point précis, le véritable challenge est de raccourcir le délai historique qui existe entre l’arrivée de ces vaccins dans les pays à revenus élevés et leur introduction dans les pays à faible revenu notamment en Afrique, et les coûts de ces vaccins qui restent élevés.

Par ailleurs, il est nécessaire de développer les capacités nationales en matière de résolution des problèmes des systèmes de santé et encourager l’utilisation des données de la recherche pour faciliter l’élaboration des politiques notamment en matière d’immunisation. Les systèmes de santé doivent reposer sur une architecture dynamique, soigneusement conçue et résolument fonctionnelle. Les programmes nationaux de vaccination devront continuer à être pleinement intégrés dans les plans nationaux de santé. Des systèmes de vaccination solides font partie intégrante d’un système de santé performant. En outre, la fourniture de services de vaccination doit continuer de servir de plate-forme pour la prestation d’autres interventions de santé publique prioritaires telles que le déparasitage, la supplémentation en vitamine A, la distribution de moustiquaires imprégnées, etc.

## Conclusion

La vaccination reste un succès indéniable de la médecine. Ces capacités de contrôle des maladies infectieuses et certains types de cancers doivent et peuvent être renforcées. Une meilleure information, la formation des personnels de santé en vaccinologie et des recherches bien ciblées sont les clés de ses futurs accomplissements. En Afrique, augmenter les capacités de prévention représente un enjeu de toute première importance pour la santé publique et son économie. Les innovations apportées par la recherche-développement aux niveaux national, régional et mondial maximisent les bénéfices de la vaccination et ouvrent bien des perspectives. Il est aujourd’hui raisonnable d’espérer que seront mis au point des vaccins efficaces contre les grands fléaux, tels que le paludisme, la tuberculose et le sida, contre l’émergence ou l’expansion de nouvelles pathologies infectieuses (en particulier Ebola et Zika).
